# Influences on the Uptake of Health and Well-being Apps and Curated App Portals: Think-Aloud and Interview Study

**DOI:** 10.2196/27173

**Published:** 2021-04-27

**Authors:** Dorothy Szinay, Olga Perski, Andy Jones, Tim Chadborn, Jamie Brown, Felix Naughton

**Affiliations:** 1 School of Health Sciences University of East Anglia Norwich United Kingdom; 2 Department of Behavioural Science and Health University College London London United Kingdom; 3 Behavioural Insights Public Health England London United Kingdom; 4 SPECTRUM Consortium London United Kingdom

**Keywords:** behavior change, health apps, mHealth, smartphone app, framework analysis, Capability, Opportunity, Motivation-Behavior model, Theoretical Domains Framework, think aloud, mobile phone

## Abstract

**Background:**

Health and well-being smartphone apps can provide a cost-effective solution to addressing unhealthy behaviors. The selection of these apps tends to occur in commercial app stores, where thousands of health apps are available. Their uptake is often influenced by popularity indicators. However, these indicators are not necessarily associated with app effectiveness or evidence-based content. Alternative routes to app selection are increasingly available, such as via curated app portals, but little is known about people’s experiences of them.

**Objective:**

The aim of this study is to explore how people select health apps on the internet and their views on curated app portals.

**Methods:**

A total of 18 UK-based adults were recruited through social media and asked during an in-person meeting to verbalize their thoughts while searching for a health or well-being app on the internet on a platform of their choice. The search was then repeated on 2 curated health app portals: the *National Health Service Apps Library* and the Public Health England *One You* App portal. This was followed by semistructured interviews. Data were analyzed using framework analysis, informed by the Capability, Opportunity, Motivation-Behavior model and the Theoretical Domains Framework.

**Results:**

Searching for health and well-being apps on the internet was described as a *minefield*. App uptake appeared to be influenced by participants’ capabilities such as app literacy skills and health and app awareness, and opportunities including the availability of apps, app esthetics, the price of an app, and social influences. Motivation factors that seemed to affect the uptake were perceived competence, time efficiency, perceived utility and accuracy of an app, transparency about data protection, commitment and social identity, and a wide range of emotions. Social influences and the perceived utility of an app were highlighted as particularly important. Participants were not previously aware of curated portals but found the concept appealing. Curated health app portals appeared to engender trust and alleviate data protection concerns. Although apps listed on these were perceived as more trustworthy, their presentation was considered disappointing. This disappointment seemed to stem from the functionality of the portals, lack of user guidance, and lack of tailored content to an individual’s needs.

**Conclusions:**

The uptake of health and well-being apps appears to be primarily affected by social influences and the perceived utility of an app. App uptake via curated health app portals perceived as credible may mitigate concerns related to data protection and accuracy, but their implementation must better meet user needs and expectations.

## Introduction

### Background

Noncommunicable diseases (eg, diabetes, heart disease and cancer as well as poor mental health) are considered key threats to global health [1] and are driven by factors such as physical inactivity, poor diet, tobacco smoking, and excessive alcohol consumption. A key global public health policy priority is to enact policies to ensure that the best possible health care is available for all [2]. In the United Kingdom, aims of the National Health Service (NHS) long-term plan [3] and priorities of UK government executive agencies such as Public Health England (PHE) are to provide a smoke-free society, to encourage healthier diets, and to improve mental health [4]. Encouraging the use of digital health interventions, such as smartphone apps, may be a cost-effective way of contributing.

Health and well-being smartphone apps can be cost-effective solutions for changing health behaviors [5,6]. Such tools can act as ideal platforms to deliver behavior change interventions [7] because of their availability, portability, and easy access [8]. Research has demonstrated early evidence of effectiveness of smartphone apps for smoking cessation [9], healthy dietary and physical activity promotion [5,10-12], weight loss [5,13,14], alcohol reduction among nondependent drinkers [15], and mental health promotion [16]. In addition, health apps can reach those resistant to seeking help in person (because of stigma) by improving access to behavior change interventions [17]. However, low uptake and poor engagement over time compromise the potential of health and well-being apps.

*Uptake* refers to the decision to select and install a health app [18]. The search for and selection of health apps tend to take place in commercial app stores such as Google Play for Android operating systems and the Apple App Store for iOS [10,19]. Thousands of health and well-being smartphone apps are available in the major app stores, a number that continues to grow [7], and the uptake of apps from commercial app stores tends to be influenced by indicators of popularity such as the app’s rank order, ratings and reviews, and the total number of downloads [19]. However, such popularity indicators are not necessarily positively associated with the effectiveness of an app [20] and may even be negatively related [21]. An associated problem with app uptake is that the vast majority of apps listed in commercial stores lack evidence about their efficacy [22] or effectiveness [23]. The need for quality marks in commercial app stores [24] and regulation of health apps and evidence for their effectiveness has been raised [16]. Better transparency in an app’s description to help people make an informed choice, including how the user’s data are handled, how the app was developed, benefits explained in lay terms, and descriptions of the app content, has been recommended [25-27].

A barrier to the uptake of evidence-informed apps is that not all apps are available to the public, or prominently displayed, via commercial app stores [22,24]. Therefore, fewer people may benefit from the high-quality tools available. Evidence-informed apps tend to be promoted within community or health care settings (often targeting a specific geographic region or country) or on curated health app *portals*. These portals are websites that present a list of selected health apps [28]. Health app portals can be government funded, such as the UK NHS’s *Apps Library* or PHE’s *One You Apps* portal, or curated by private organizations, such as *App Script* by IQVIA in the United States, the United Kingdom, and the United Arab Emirates; the *MyHealthApps* by PatientView’s in Europe and the United Kingdom; or *ORCHA* Health in the United Kingdom. These organizations can lend credibility to and have the potential to promote the uptake of selected health apps [29] by providing a list of safe; evidence-informed; tested; and, where possible, clinically effective health apps for the general public to choose from.

Research has focused on the identification of factors that influence the uptake of health apps in commercial app stores. There is an urgent need to explore whether the general public would be willing to use curated health app portals, which could improve the uptake of evidence-informed health and well-being apps [18]. Despite this need, little is known about the views on curated health app portals. This study aims to explore potential users’ views on factors influencing the uptake of health apps in general and on curated health app portals in particular using think-aloud and interview methodology.

### Theoretical Framework

**Figure 1:**
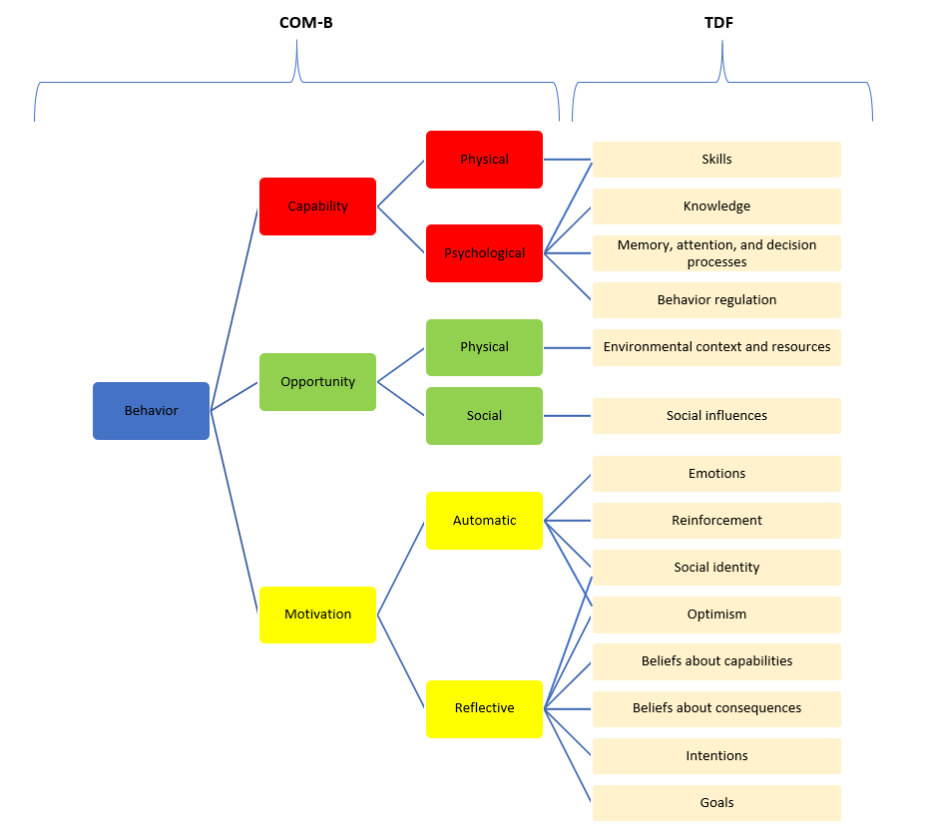
A visual representation of mapping the TDF onto the COM-B model. COM-B model: Capability, Opportunity, Motivation-Behavior model; TDF: Theoretical Domains Framework.

The Capability, Opportunity, Motivation-Behavior (COM-B) model [30] offers a comprehensive framework for understanding behaviors. In the context of this study, the behavior of interest is the uptake of health and well-being apps. The model proposes that behavior arises because of the interaction of three components: capability (physical and psychological), opportunity (physical and social), and motivation (automatic and reflective). The Theoretical Domains Framework (TDF) [31], which contains 14 domains that can be mapped onto the components of the COM-B model, was also used. Together, the COM-B model and the TDF allow for a detailed analysis of data and identification of key factors influencing uptake in general and on curated health app portals in particular (Figure 1) [18].

### Aims

This qualitative study applied a theoretical framework informed by the COM-B model and TDF to explore (1) factors influencing potential users’ uptake of health and well-being smartphone apps through searching on the internet and (2) their views on available curated health app portals.

## Methods

### Study Design

This study elicited views and preferences of a sample of members of the public. The Consolidated Criteria for Reporting Qualitative Research checklist guided the design of the study [32] (checklist given in Multimedia Appendix 1). The think-aloud methodology [33] was applied to collect real-time data about health app selection on the internet and involved asking participants to verbalize their thoughts and impressions throughout the selection process. The researcher intervened only when a prompt was considered necessary (eg, during silent moments, asking questions such as “What are you thinking now?”). Following the think-aloud tasks, follow-up questions were asked to better understand the statements or utterances made during the tasks. Finally, semistructured interviews were conducted. The think-aloud tasks and the topic guide were informed by stakeholder consultation, which included views and opinions of lay persons (patient and public involvement representatives) and expert opinions of policy makers of this study. The study protocol was preregistered on the Open Science Framework [34]. The Faculty of Medicine and Health Sciences Ethics Committee at the University of East Anglia approved this study (reference number: 201819-089). The collected data are stored following the European Union General Data Protection Regulation and the University of East Anglia Research Data Management Policy. The data were anonymized, and all personal identifiers were removed. All participants read the participant information sheet and provided consent before participating in the study.

### Participants and Recruitment

Participants were recruited through paid advertisements on Facebook. Adults in the general population were eligible if they were 18 years or older; were able to provide consent; owned a smartphone; would consider using a smartphone app to change their behavior in the future; and were able to attend an interview in Norwich, England, where the work took place. As a standard practice in qualitative research, the aim of this study is to gain a better understanding of the phenomenon of interest and to increase the coverage of perspectives rather than to recruit a population-representative sample [35]. Therefore, purposive sampling was used to promote the diversity of the sample (ie, age, gender, ethnicity, educational level, and employment) [36]. This included targeted advertisements on Facebook and the selection of participants to ensure the diversity of the sample. A total of 114 individuals responded to the Facebook advertisements and read a brief participant information sheet and completed the screening questionnaire. Of the 38 participants invited to an interview, 14 did not respond and 24 agreed to participate. Of these 24 participants, 6 were canceled for various reasons.

### Procedure

Before completing the online screening survey, participants were asked to read a brief participant information sheet describing the study. After reading and agreeing to participate, participants were asked to complete an online questionnaire to assess their eligibility and to collect descriptive data (Multimedia Appendix 2). Data were collected on age; gender; ethnicity, measured using the Office for National Statistics’ index; level of education; employment status; whether they had ever used health or well-being apps; whether they currently use a health or well-being app; last time they had downloaded an app; and frequency of app use. Participants who met the inclusion criteria were sent an email with a comprehensive participant information sheet and invited to participate in the interview. On the day of the interview, the interviewees received a printed copy of the participant information sheet, and written consent was obtained.

**Figure 2:**
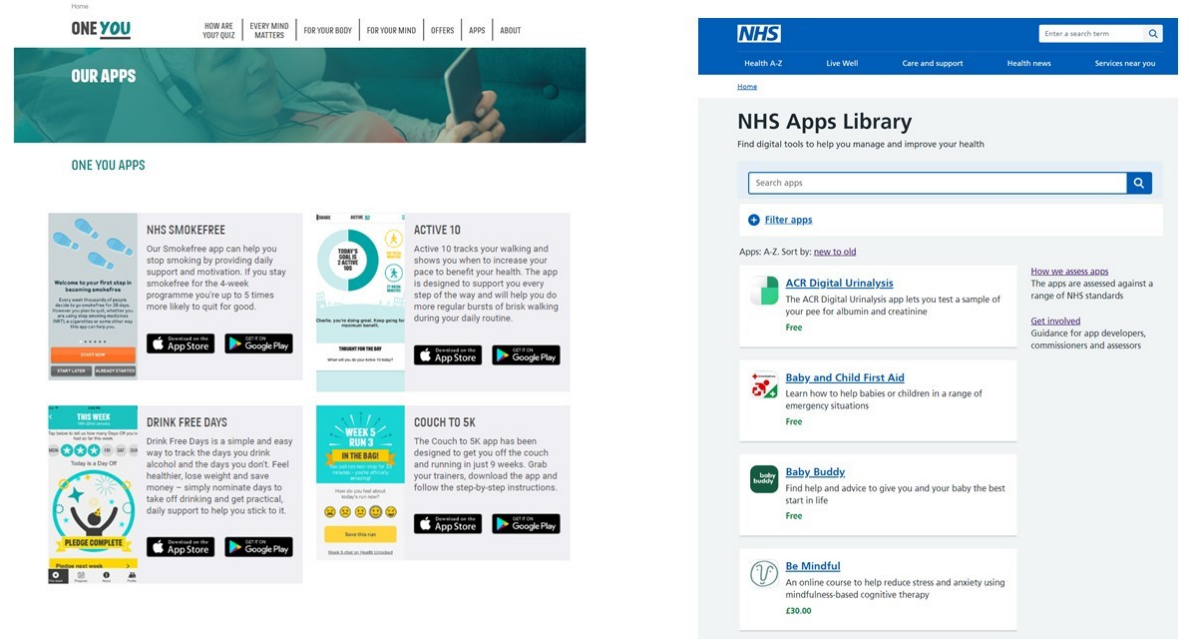
Screenshot of the Public Health England’s ‘One You Apps’ portal and the ‘NHS Apps Library’.

Face-to-face interviews were conducted between July and August 2019 and took place at the University of East Anglia (n=17) or participants’ homes in Norwich (n=1). The interviews were conducted by a single female researcher (DS), and no one else was present during the sessions. Each session started with a think-aloud exercise, with participants being instructed on how to verbalize their thoughts. First, they were asked to perform a search for an app they would potentially use to change the health behavior of their choice. They had a choice of using either a study laptop or their smartphone. Second, the researcher asked them if they were familiar with curated app portals. If they were not, DS briefly explained the principle and asked them to repeat the search using the *NHS Apps Library* and the PHE’s *One You Apps* curated health app portals (Figure 2). During the think-aloud sessions, positive reinforcement using verbal (eg, “You are doing great” and “Right”) and nonverbal (eg, nodding) communication was used to encourage participants to continue to express their views. In quiet moments, prompts were used (eg, *“*What are you thinking now?” and “Tell me what is on your mind”). Following the think-aloud task, questions regarding their experience with the uptake of and engagement with apps were asked (the topic guide is given in Multimedia Appendix 3). The sessions lasted between 26 and 63 minutes. Participants received a US $27.50 (UK £20) gift voucher as compensation for their time.

### Data Analysis

The sessions were audio recorded and transcribed verbatim by an external company. The transcriptions were checked for accuracy by the researcher undertaking the interviews. The data were analyzed using framework analysis following the stages of familiarization, identification of thematic framework, indexing, charting, mapping, and interpretation [37]. To ensure rigor, trustworthiness, and consistency, a percentage of randomly selected transcripts (2/18,15%) were independently coded by the second author (OP). The deductive thematic framework based on TDF was refined iteratively through repeated discussions with the second author (OP), and any discrepancies were resolved through discussion with the senior author (FN). Indexing was completed by the first author (DS) using QSR NVivo 12. The data were charted, and the responses were grouped according to the finalized thematic framework. During mapping and interpretation, the grouped data were examined by DS to identify patterns. During mapping, identified factors were classified according to their organic position rather than what they affect (eg, an opportunity factor may indirectly influence the behavior by increasing the motivation for uptake of a health app and influencing it directly). To aid comprehension of the findings for uptake in general and on health app portals in particular, data were analyzed and presented separately for these 2 topics.

### External Validity

To enhance the credibility and trustworthiness of the results [38], 30% (6/18) of participants were randomly selected and requested via email to provide feedback on a document with a summary of the findings and conclusions (*member checking*). They were asked whether they recognized their opinions and whether they agreed with the interpretation of the findings. A total of 2 participants responded to our request and confirmed that their opinions had been captured. In one case, our email was not delivered.

### Reflexivity

The researchers involved in this study are mixed methods researchers with experience applying the COM-B model and TDF to qualitative data. She disclosed her research interest to participants on the day of the interview, and no previous relationship was established between her and participants. The interviews were conducted by the lead author, a PhD candidate who has undertaken extensive training in the collection and analysis of qualitative data. Participants were encouraged to share their thoughts (both positive and negative) and to be honest. The interviewer felt that good rapport was built with the interviewees, and most participants (n=16) expressed their interest in learning more about the findings of the research. Field notes and a research journal were kept during data collection.

## Results

### Participant Characteristics

A total of 18 participants completed the interview. The average age of participants was 43 (SD 14) years, 50% (n=9) were female, 78% (n=14) were of White British ethnicity, 72% (n=13) were employed full time, 11% (n=2) had postgraduate qualifications, 94% (n=17) had used health apps before, and 61% (n=11) were using health apps at the time of the interviews, out of which 73% (n=8) reported daily health app use. Most participants were interested in changing more than one behavior (eg, losing weight, getting more active, and managing mood), and only 16% (n=2) of participants expressed a desire to change only one behavior. Participants’ characteristics are presented in Multimedia Appendix 4.

A total of 2 participants were satisfied with the app they were already using and did not wish to take part in the think-aloud exercise to look for a different app. The remaining 16 participants searched for apps targeting physical activity (n=6), weight management (n=4), mood and mental well-being (n=3), smoking cessation (n=1), alcohol reduction (n=1), and sleep (n=1).

The findings pertaining to factors relevant for both the uptake of health apps and views on curated health app portals are presented under the components of the COM-B model. Higher order themes and subthemes informed by the COM-B model and TDF are reported in Table 1.

**Table 1:** Factors influencing uptake of health apps in general and on health app portals mapped onto the components of the Capability, Opportunity, Motivation-Behavior model and Theoretical Domains Framework constructs.

COM-B^a^ component and TDF^b^ construct and identified factor				Uptake in general		Uptake on health app portals
**Physical capability**						
	**Skills**					
		App literacy	Technological competency		—^c^	
**Psychological capability**						
	**Knowledge**					
		Health awareness	General health consciousness or having family members diagnosed with a condition or disease or concerns regarding a behavior or health outcome		—	
		App awareness	Knowledge of the existence of health and well-being apps		Knowledge of the existence of health and well-being apps listed on health app portals	
		User guidance	—		Instructions on how to effectively use a health app portal	
		Health information	—		Educational information related to health and well-being	
	**Memory, attention, and decision processes**					
		Cognitive load	—		The manner in which apps are presented on the portal The complexity of the search or to access a relevant health app	
**Physical opportunity**						
	**Environmental resources**					
		Availability	The ability to use a smartphone anytime, anywhere Availability of an app on all major commercial app stores		—	
		Portal tailored to individuals’ needs	—		Personalized listing of apps targeting age, gender, and health condition	
		Cost of an app	Low cost and apps that are free for users		Low cost and apps that are free for users	
		Esthetics	The look and design of an app		User-friendly and design-related characteristics of the portal	
**Social opportunity**						
	**Social influences**					
		Social influences	The importance of reviews and ratings in the commercial app stores and apps promoted as ”editor’s choice” Identified credible sources: apps developed or endorsed by trusted app developers, organizations, or universities or promoted by respected celebrities (eg, athletes) Recommendations received from health practitioners or from friends and family		Health app portals perceived as credible sources Recommendations of health app portals needed mainly in primary care Clarity about the recommended apps on health app portals Explanations about any required GP^d^ referral	
**Reflective motivation**						
	**Beliefs about capabilities**					
		Perceived competence	Apps preferred over face-to-face intervention when the user feels that they can engage with the app on their own		—	
	**Beliefs about consequences**					
		Time efficiency	The ability of a health app to be interacted with a minimum amount of time		—	
		The perceived utility of the app	Discrepancies between what users are looking for and what the app offers, characterized by a relevant title, description, pictures, adaptation to individual characteristics, and users’ previous experience with health apps		Discrepancies between what users are looking for and what the app listed on health app portal offers, characterized by a relevant title, description, and pictures	
		Perceived accuracy	The perceived effectiveness of apps before the selection of an app		Potential app users’ perceived effectiveness of apps listed on health app portals	
		Data protection	Concerns regarding the handling of personal data		Concerns over the handling of personal data	
	**Intentions**					
		Commitment	The level of commitment when deciding to download a health app		—	
	**Social identity**					
		Social identity	Identity related to app use (eg, trends and gender specificity)		Identity related to app use (eg, feeling like a “patient”)	
**Automatic motivation**						
	**Emotions**					
		Positive	Triggered by curiosity in trying a health app, and by the time efficiency characteristic of an app as opposed to face-to-face interventions, and being provided by a credible source		Triggered by curiosity in choosing a behavior change tool from a curated health app portal and from a credible source	
		Negative	Triggered by lack of availability on all major app stores Preferred over a face-to-face intervention if feeling anxiety (eg, caused by an unhealthy behavior or unhealthy state) and pressurized (to succeed or show progress)		Triggered by lack of search features on the portal or when the search yields irrelevant results; when an app requires GP referral without further explanation or when an app is only available in one major app store	
		Mixed	Triggered by the esthetics (design) of the apps and by adaptation to individual characteristics (judged by the title, description, pictures, and gender specificity)		Triggered by the esthetics and features of the portal and the perceived utility of the apps	

^a^COM-B: Capability, Opportunity, Motivation-Behavior.

^b^TDF: Theoretical Domains Framework.

^c^Not available.

^d^GP: general practitioner.

### Factors Influencing the Uptake of Health and Well-being Apps

Half of the participants who agreed to search for a health app (n=8) used Google Search as their first choice to find a suitable app, whereas the other half opened a commercial app store. The latter search among hundreds of available apps was described by most participants as difficult or a “minefield” (P2, P4, and P6). One participant described this task as being “far more complicated than I thought it would be*”* (P2). By the end of this exercise, only 3 participants found an app that they were willing to download and engage with further to change their behavior.

#### Capability Factors Related to the Uptake of Health and Well-being Apps in General

Participants who presented a higher level of technological competency were able to better navigate on their phones, thus highlighting that app literacy skills are necessary when selecting a health app. One participant, who had never used a health app before, showed signs of technical difficulties (ie, lack of skills) during the think-aloud exercise while searching for an alcohol reduction app in a commercial app store:

I wouldn’t know how to do that [refining the search to find a suitable app].

P12

In addition, 2 participants expressed their concern toward the older generation and stated that training should be provided for those with insufficient technological and app literacy skills:

My nanny is diabetic and if there was an app to help her with her diabetes, then I’m sure she would be happy to use it but it’s just someone would need to explain it to her.

P18

All participants expressed their decision to look for an app for health reasons, such as getting healthier or preventing illness. This included reasons of being diagnosed, or having a family member diagnosed, with a medical condition (eg, diabetes and high blood pressure) or concerns of the negative effect a current behavior may have (eg, smoking and alcohol consumption) to better manage or improve their mental health (eg, anxiety and self-confidence) and general well-being (eg, sleep quality):

I’m trying to avoid having type 2 diabetes, or getting it, so there’s a background, my mother, in my family, there’s a heart conditions background, which is why I’m really wanting to do something about my health.

P3

Although most participants were aware of the existence of some apps, 3 participants were surprised by the existence of health apps for smoking cessation and mental health issues:

It didn’t cross my mind that I could use an app for stopping smoking, so it is new.

P16

#### Opportunity Factors Related to the Uptake of Health and Well-being Apps in General

Some participants expressed their preference to look for a health app as a digital behavior change intervention instead of a face-to-face intervention because of the availability and low cost of an app. However, concerns around widening inequalities were raised by one participant who showed signs of worry about the limited access to digital aids for individuals living in deprived areas:

So if they [people living in deprived areas] do not have the smart phone, they won’t be able to use it, so it’s not going to work, is it? It’s what happened with the Universal Credit, so it’s not going to work. I mean issue everyone a smart phone.

P16

A few participants highlighted the importance of the availability of health apps in both major commercial app stores (Apple App Store and Google Play), not just one or the other.

Most participants stated that apps should be available at no cost. Only 6 participants expressed their willingness to pay a small fee for an app if, for example, it would be “almost life-changing” (P4) or if it would include online professional support.

The specific design and color scheme preferred by participants appeared to be unique and dependent on the individual’s taste. However, the majority were looking for a *simple* looking app.

Social influences appeared to be one of the core factors that shaped the selection of apps for all participants during the think-aloud exercise. This includes ratings and reviews of the app, the credibility of the source of the app, and recommendations of apps received from others. Within app stores, most participants described looking at the star ratings and the number of downloads of each app and whether the apps were listed as an *editor’s choice*. A total of 3 participants acknowledged that reviews were subjective, and they still reported feeling influenced by the ratings of the app. In addition, 2 participants reported that they were skeptical of the reviews, which they believed may have been paid for, and that reviews are not enough, as more information is necessary to make an informed choice:

You know, so you're having to make all these judgements about people’s reviews and then you know deep down that the reviews might be paid for and, you know, it’s a bit of a minefield which is why I would only take a free sample and then see if it works for me.

P6

A credible source was also important. Apps developed or recommended by trusted organizations or respected celebrities seemed more appealing to all participants. Participants who used Google Search to find an app aimed to look for websites they were familiar with or had used before or for websites that would post “Top 10 apps for...” type of articles. In addition, word of mouth was another source of social influence:

I see two different specialists, I have a lung problem as well and I see a lung specialist at a hospital near me and she said to me, the best thing that I could do, which was downloading the Couch to 5k app.

P14

#### Motivational Factors Related to the Uptake of Health and Well-being Apps in General

Health or well-being apps were preferred over face-to-face options because participants reported feeling competent by changing their behavior through the use of an app, requiring less time commitment and avoiding the anxiety and pressure of interacting with others. Time appeared to be a particularly valuable resource for all participants, and they believed apps to have this advantage.

Another core factor in the selection of an app was the way users perceived its utility. This was based on 2 aspects. First, they appeared to judge how the app is adapted to the individual by reading the title and description of the app and by looking at pictures (ie, screenshots). A total of 12 participants reported the need for sufficient information about an app to make an informed choice:

I would definitely judge more from the pictures more than anything and I think that just nowadays everyone does, is you get an idea of the app from the pictures. (...) I mean I think when you see an older person on a picture and you’re a lot younger, it makes you think, I mean it’s the wrong think to think but it makes you think maybe it’s not for me.

P7

Second, it seemed that 12 participants relied on their past experiences with health apps. Whether those experiences were positive or negative may have shaped their beliefs about health apps in general:

So that’s why My Fitness Pal is the first app that I’ve ever had that’s actually worked.

P9

In addition, 7 participants expressed skepticism about the accuracy and effectiveness of some apps (eg, mental health apps), and concerns about data protection were mixed:

These mindful ones, I’ve never downloaded one and I’m sceptical.

P17

Participants mentioned that commitment to the behavior change would influence uptake and future engagement:

So I think the committed ones seek out the ones that are the right ones for them, the best ones, rather than necessarily the trendy ones.

P4

Participants’ social identities also shaped their selections. Many reported that they did not wish to select apps that promoted groups they did not seem to fit in with (eg, athletic body image or individuals of the hipster subculture):

They’ve got a kind of hipster bloke and now they’ve got a kind of sexy female image with tattoos down her arm, sexy, trendy, female image. Okay, so they are obviously aiming at younger, sort of people in their twenties and thirties, yeah, another sexy female image. It’s quite interesting isn’t it, I’m looking at the images and not the words and getting a sense, is this for me, middle aged, well older woman?!

P6

Curiosity, defined here as a desire to learn something, was the only stand-alone positive emotion and appeared to positively influence the uptake of health apps for many participants:

I thought out of curiosity I’d have a look, so I just typed in quit smoking in Google play store and there’s hundreds of apps from various people with varying degrees of credibility, and they all were pretty similar to be honest.

P13

Apps linked to a credible source were important, with people unimpressed when an app was not available on all major app stores.

### Views on Curated Health App Portals

None of the participants spontaneously used a curated portal. Curated portals were then introduced to the participants, but none were previously aware of them. Curated health app portals were appealing to all participants, and they believed the portals would be likely to engender trust. However, searching for a health app on the NHS Apps Library and the One You App portal was a generally disappointing experience. Only 2 participants chose a health app from a health app portal (One You Apps), whereas the rest of the participants decided to continue the search in commercial app stores.

#### Capability Factors Related to the Uptake of Health and Well-being Apps on Health Portals

All participants had heard of widely advertised apps (eg, Couch to 5k), but none were aware of the existence of curated health app portals before participating in this study:

I think they’re brilliant [apps on health app portals]; I didn’t know they existed.

P11

Navigating on the NHS Apps Library seemed easy for some. However, a few participants mentioned that a user guide or help section would be a useful added feature of the portal. Two participants reported that they did not find it easy to use the filter features, and in many cases, they felt the search yielded irrelevant results (eg, while searching for a physical activity app, the results also listed apps for mental health). A few participants reported that navigating on curated app portals was difficult, characterized as “cumbersome” (P4, P12):

It’s not clear, it’s suggests that they are independent apps but maybe they should have some guidelines about design, you know, of their sort of landing pages.

P6

#### Opportunity Factors Related to Uptake of Health and Well-being Apps on Health Portals

All participants indicated that they would want a portal tailored to their needs, with categories related to their gender, age group, and medical conditions they may have:

So something like that, this is suitable if you’re over 65, this would be more suitable for you if you’re under 40 or with these ones that you don’t have to go and see your GP, that you can pay for, if you have any concerns, visit your GP or speak to a health professional because some people don’t have that common sense.

P14

Participants had different opinions about the layout of these portals. Some liked the NHS Apps Library design better, with simple colors, whereas others enjoyed the more colorful One You App portal. Most participants felt that a fusion between these 2 designs (the searchability and filters of the NHS Apps Library and the look and presentation of the One You App portal) and a better functionality would create the ideal curated health app portal:

Why they are not combined?

P8

Although many participants expressed their wish to access apps for free, a few participants were more open to pay for an app that was listed on a curated health app portal:

This is fabulous, and I’d be much more inclined to pay money. This is really, really good.

P6

Participants found the NHS and PHE trustworthy and believed that these portals would provide safe and effective digital aids. Some indicated a desire to receive further recommendations for using these portals from their primary care physicians:

If GPs knew that they could say “well this could help you” I’m sure that they would recommend it to people.

P11

However, they also wanted to avoid putting unnecessary pressure on general practitioner (GP) practices:

You’ve got “free but requires GP referral” and when you’re thinking the NHS is under so much financial strain and pressure at the moment, why do I need a GP referral to obtain an app?

P2

In addition, the One You App portal lists a few apps that are recommended, but participants expressed their confusion and lack of clarity regarding why some apps are *recommended* and by whom.

#### Motivation Factors Related to Uptake of Health and Well-being Apps on Health Portals

While searching on curated health app portals, none of the participants expressed signs of concern about data protection and accuracy of apps, although 2 participants reported that they would want to read more about how these apps were developed and tested:

How long it takes, how many sessions and the fact that it’s been tested in clinical trials and evaluated by NICE which, to me, is probably quite an important thing.

P1

Social identity was also important. Some participants had identified themselves as individuals living with a medical condition. These participants were keen to look for an app that targets the behavioral change of individuals with preexisting medical conditions. Others stated that they do not wish to feel “like a patient” (P7) and seemed reluctant to continue the search on a curated health app portal:

So it would be nice to have one specific for maybe people with medical problems or age-related problems, etc.

P15

## Discussion

### Principal Findings

Online searches for health and well-being apps were found to be difficult. Factors influencing the uptake of health apps were mapped using the COM-B model and TDF. We found that social influences and participants’ beliefs about consequences (the perceived utility of the app) are key factors influencing the uptake of health apps. This conclusion was based on the frequency and salience of the themes that occurred during the interview. Curated health portals were found to be appealing despite the lack of awareness of their existence. However, the way apps are currently presented on these portals did not meet users’ needs because of a lack of certain features, such as lack of tailoring to the user’s requirements.

In line with previous research, the findings revealed the importance of the capability and opportunity factors, such as app literacy skills; health awareness and app awareness; esthetics of an app; low cost of an app; reading reviews and checking ratings; credible sources; and recommendations of apps from others, including health professionals [18,22,39,40]. Interestingly, the perception of the cost of an app appeared to be related to the perceived utility and credibility of the source. Although at the start, some participants were against paying for apps, the more useful an app was perceived, the more inclined participants felt to pay a fee. This phenomenon was observed for apps listed on health app portals, which were considered a credible source. More importantly, unlike apps listed on commercial app stores, there was implied trust in apps listed on curated health app portals by participants. In addition, some health apps are not available for downloading in both commercial app stores. Participants found it disappointing that some apps were only available for iPhone users. This is in line with previous research that found that out of 18 investigated health apps, only one-third were available to download on both major commercial app stores [28].

In terms of motivational factors, we found that perceived utility included aspects related to individuals’ perceptions about the presentation of an app and their previous experiences with health apps. Together, these shaped the way participants judged the usefulness of an app. This characterization underlines the need expressed by others previously for a better way to present health apps through a description that would lead to an informed choice (eg, the content of the app) [25-27] and potentially positively affect other motivational factors, such as the accuracy of an app and data protection [41]. Notably, concern about data protection and the accuracy of a health app was minimal when participants navigated on health app portals as opposed to commercial app stores.

There is a need to understand what design aspects generate positive or negative emotions and for whom. Emotions are powerful drivers of a behavior, which affects decision making (eg, app uptake) [42]. A key emotion identified in this study directly influencing the uptake was curiosity. However, this study emphasized the importance of positive emotions triggered by, for example, the credible source of an app and negative emotions triggered by restriction of information (eg, lack of understanding of the necessity of GP referral to download an app). Taking these factors into consideration may lead to better uptake with such tools.

Uptake and engagement are connected. Engagement without uptake is not possible, and uptake without taking into consideration the factors that are important for engagement is impractical. Some factors might influence both uptake and engagement; for example, our research suggests that the perceived utility of an app is one of the main factors for uptake. However, a previous study found that perceived utility was a predictor of engagement with an alcohol reduction app [43]. Therefore, where possible, uptake and engagement should be considered together as 2 linked constructs.

### Strengths and Limitations

The main strength of this study lies in its methodology. Given that the aim of this study is to explore uptake with health apps and by applying a user-centered approach, the think-aloud methodology was the appropriate technique to use [33,44] as it will minimize recall bias. Involving stakeholders—patient and public engagement representatives and policy makers—in the design of the research enhances scientific rigor. The purposive sampling technique adopted enabled the recruitment of a wide range of participants that included the same number of females and males and having different levels of education and employment status, and the sample overrepresented ethnicity relative to local rates. The use of the COM-B and TDF to guide the data analysis is another strength of this study.

This study had several limitations. First, asking participants to perform the think-aloud task under observation may not be fully analogous to how they would perform a search when on their own. Second, some identified factors were difficult to define and describe because of the lack of specificity of the description provided by participants. These include esthetics of apps, often described vaguely (*nice* and *elegant*) and the cognitive load associated with engagement with these (*easy to use*). Third, for a qualitative research study exploring such a broad topic, we felt that information saturation was reached; however, it is possible that additional participants with more varied characteristics would have allowed us to identify additional concepts. Finally, during external validation, a randomly selected subsample of participants was asked via email to provide feedback on the summary of the findings. A total of 50% (3/6) of participants did not reply, and it is unclear whether these participants ignored our request or did not agree with the interpretation of the results.

### Implications for Research, Policy, and Practice

This study has important implications for stakeholders in public health and policy makers who target prevention and health promotion using digital technologies and governmental bodies and trusted health organizations that provide curated health app portals. Low awareness, low app literacy skills, lack of availability on all major app stores, and lack of recommendation in primary care were identified as factors limiting the uptake of health apps in general and on curated app portals. These factors are important for improving the uptake of health apps. Selection was described as difficult. Therefore, there is a need for public guidance on how to identify evidence-based tools [18,22] and for health practitioners to promote and advise their patients on how to select appropriate health and well-being apps [40]. Raising awareness of such tools through both online and offline promotion channels might provide better access to effective apps.

Our findings could also help developers to reconsider the ways in which apps are currently presented on commercial app stores and app portals, which might, in turn, increase the uptake of evidence-informed health apps. The idea of selecting an app from a health app portal was appealing to all participants, although individuals’ needs were not met. These findings describe essential barriers and facilitators related to participants’ capability, opportunity, and motivation to take up health and well-being apps. For example, app descriptions and presentations that better align with individuals’ needs may increase the uptake of health apps on health app portals. These findings can also be used to inform the development of interventions that specifically aim to promote the uptake of and engagement with evidence-informed health and well-being apps, a priority within the NHS long-term plan (ie, *digital first*). By targeting the identified psychological influences on app uptake through further interventional work, organizations that provide app portals (eg, the NHS and PHE) should be able to increase their impact by helping people to better select appropriate apps. A summary of the recommendations for policy makers, providers, and developers is presented in Textbox 1.

**Recommendations for policy makers, industry, health care providers, and app developers based on the Capability, Opportunity, Motivation-Behavior model for a better uptake of health and well-being apps.**

Capability

- Improve app literacy skills, with a focus on older and marginalized populations, and continue working toward reducing the digital divide (eg, through the use of an outreach approach to target older, migrant, and homeless populations).
- Increase awareness of effective health apps and curated health app portals through promotion online and offline in primary care, mass media, and public spaces.
- Provide guidance on how to use a health app portal (eg, through incorporating an extensive help section) and additional physical and mental health–related evidence-based papers.
- Promote reduced cognitive load on curated health app portals (eg, through the use of images and short app descriptions).

Opportunity

- Ensure evidence-informed apps are available for free or at a low cost to everyone.
- Make apps available on all major app stores simultaneously.
- Offer the possibility to tailor the health app portal to target certain demographics (eg, apps for physical activity for women aged 60 years or more).
- Offer apps at low cost and provide explanation for those that require referrals and justifications for the cost of paid apps on curated health app portals.
- Collaborate with interaction design experts and end users to enhance the esthetics of health app portals.
- Promote evidence-informed apps via trusted organizations and provide information on how the apps were developed and tested.
- Encourage health professionals and practitioners of promotion of evidence-informed health apps and health app portals.

Motivation

- Provide relevant and realistic titles and avoid general app descriptions. Descriptions should be short but must contain details of what the app offers and how it is able to help the user.
- Provide pictures of the app (eg, screenshots) and avoid pictures that promote an unrealistic body image.
- Provide information about the accuracy and effectiveness of the app (eg, details about development and developers) and how users’ data are handled.
- Take into account users’ emotions about certain features by constantly involving the users in the development of health apps.

### Future Research

Future research is needed to minimize factors limiting uptake, such as low awareness, low app literacy skills, and a lack of recommendations in primary care. Our results suggest that there is a need to better tailor the design and content of health app portals to better meet individuals’ needs. However, the mixed views on specific app designs indicate that more research is needed to investigate whether there are general design principles that are missed and could be followed to accommodate the majority of people or whether better tailoring and/or adaptive interventions should be considered instead. Future research may also want to consider comparing curated health app portals developed by private organizations with those developed by governmental bodies to investigate whether portal design–related features are considered less or more important than credibility and trust in apps listed on them. Experimental research is needed to assess whether there is a trade-off between credibility, social influences, and perceived utility of the apps presented on curated health app portals. Furthermore, with a growing concern around widening inequalities [45], solutions should be focused on reducing the digital divide and health inequalities that may appear as a result of the financial constraint of owning a smartphone and lack of sufficient app literacy skills.

### Conclusions

Among the factors mapped under capability, opportunity, and motivation components of the COM-B model, social influences and the perceived utility of an app appear to be the core factors influencing uptake in general and on curated health app portals. Curated app portals are considered trustworthy and serve as a credible source for apps; however, there is disappointment with their current implementation of these portals. Uptake of health and well-being apps on health app portals, as opposed to uptake in general, appears to help address people’s concerns regarding data protection and the accuracy of apps. Health organizations that develop app portals may consider targeting the factors identified across the COM-B and TDF as part of additional experimental work, as this could help to increase impact through better selection of appropriate health apps.

## Appendix

Multimedia Appendix 1 Consolidated criteria for reporting qualitative studies: 32-item checklist.

Multimedia Appendix 2 Screening questionnaire.

Multimedia Appendix 3 Topic guide for the interviews.

Multimedia Appendix 4 Study participants’ characteristics.

## Abbreviations

COM-B: Capability, Opportunity, Motivation-Behavior
GP: general practitioner
NHS: National Health Service
PHE: Public Health England
TDF: Theoretical Domains Framework
