# Supplementary material for: Influences on the Uptake of Health and Well-being Apps and Curated App Portals: Think-Aloud and Interview Study
Source: JMIR Mhealth Uhealth. 2021 Apr 27;9(4):e27173. doi: 10.2196/27173 (PMC8114158; doi:10.2196/27173)
Supplement: Multimedia Appendix 1 [file mhealth_v9i4e27173_app1.pdf]

Multimedia Appendix 1.

Consolidated criteria for reporting qualitative studies (COREQ): 32-item checklist

Developed from:
Tong A, Sainsbury P, Craig J. Consolidated criteria for reporting qualitative research (COREQ): a 32-item checklist for interviews and focus groups. *International Journal for Quality in Health Care*. 2007. Volume 19, Number 6: pp. 349 – 357

No. Item	Guide questions/description	Reported on Page #
**Domain 1: Research team and reflexivity**		
Personal Characteristics		
1. Inter viewer/facilitator	Which author/s conducted the inter view or focus group?	Pg. 5
2. Credentials	What were the researcher's credentials? E.g. PhD, MD	Pg. 1 (title page, list of authors), Pg. 6
3. Occupation	What was their occupation at the time of the study?	Pg. 16
4. Gender	Was the researcher male or female?	Pg. 5
5. Experience and training	What experience or training did the researcher have?	Pg. 6
Relationship with participants		
6. Relationship established	Was a relationship established prior to study commencement?	
7. Participant knowledge of the interviewer	What did the participants know about the researcher? e.g. personal goals, reasons for doing the research	Pg. 6
8. Interviewer characteristics	What characteristics were reported about the inter viewer/facilitator? e.g. Bias, assumptions, reasons and interests in the research topic	Pg. 6
**Domain 2: study design**		
Theoretical framework		
9. Methodological orientation and Theory	What methodological orientation was stated to underpin the study? e.g. grounded theory, discourse analysis, ethnography, phenomenology, content analysis	Pg. 5
Participant selection		
10. Sampling	How were participants selected? e.g. purposive, convenience, consecutive, snowball	Pg. 5

11. Method of approach	How were participants approached? e.g. face-to-face, telephone, mail, email	Pg. 5
12. Sample size	How many participants were in the study?	Pg. 5
13. Non-participation	How many people refused to participate or dropped out? Reasons?	Pg. 5
Setting		
14. Setting of data collection	Where was the data collected? e.g. home, clinic, workplace	Pg. 5
15. Presence of non-participants	Was anyone else present besides the participants and researchers?	Pg. 5
16. Description of sample	What are the important characteristics of the sample? e.g. demographic data, date	Pg. 7; Multimedia appendix 1
Data collection		
17. Interview guide	Were questions, prompts, guides provided by the authors? Was it pilot tested?	Multimedia appendix 4
18. Repeat interviews	Were repeat inter views carried out? If yes, how many?	N/A
19. Audio/visual recording	Did the research use audio or visual recording to collect the data?	Pg. 6
20. Field notes	Were field notes made during and/or after the inter view or focus group?	Pg. 7
21. Duration	What was the duration of the inter views or focus group?	Pg. 6
22. Data saturation	Was data saturation discussed?	Pg. 15
23. Transcripts returned	Were transcripts returned to participants for comment and/or correction?	Pg. 6
**Domain 3: analysis and findings**		
Data analysis		
24. Number of data coders	How many data coders coded the data?	Pg. 6
25. Description of the coding tree	Did authors provide a description of the coding tree?	Table 2.
26. Derivation of themes	Were themes identified in advance or derived from the data?	Pg. 6
27. Software	What software, if applicable, was used to manage the data?	Pg. 6
28. Participant checking	Did participants provide feedback on the findings?	Pg. 6
Reporting		
29. Quotations presented	Were participant quotations presented to illustrate the themes/findings? Was each quotation identified? e.g. participant number	Pg. 10-13
30. Data and findings consistent	Was there consistency between the data presented and the findings?	Pg. 8 -13
31. Clarity of major themes	Were major themes clearly presented in the findings?	Table 1.
32. Clarity of minor themes	Is there a description of diverse cases or discussion of minor themes?	Table 1.
