# Supplementary material for: Influences on the Uptake of Health and Well-being Apps and Curated App Portals: Think-Aloud and Interview Study
Source: JMIR Mhealth Uhealth. 2021 Apr 27;9(4):e27173. doi: 10.2196/27173 (PMC8114158; doi:10.2196/27173)
Supplement: Multimedia Appendix 2 [file mhealth_v9i4e27173_app2.pdf]

Multimedia Appendix 2.

Screening questionnaire

Question	Response Options
How old are you?	Enter free text
Are you able to travel to Norwich for the interview?	(1) Yes<br>(2) No
Do you own a smartphone with Internet access and capable of running apps?	(1) Yes<br>(2) No
Which of the following best describes you?	(1) I would like to stop smoking<br>(2) I would like to drink less or stop drinking<br>(3) I would like to lose weight to get healthier<br>(4) I would like to do more physical activity<br>(5) I sometimes feel down or depressed, and I would like to feel better<br>(6) I sometimes have anxiety, and I would like to feel better<br>(7) I would like to improve my mood<br>(8) Other: [Enter Free Text]<br>(7) None of these describes me
Have you used a health or wellbeing smartphone app to help you become healthier or to feel better?<br>*(Examples of health or wellbeing smartphone apps: apps that can help you quit smoking, drink less, being more active, losing weight, become less depressed, become less anxious, improve your mood)*	(1) Yes<br>(2) No
If yes, what was the name of the health or wellbeing smartphone app(s) that you have used?	Enter free text
Are you currently using a smartphone app to help you become healthier or to feel better?	(1) Yes<br>(2) No
If yes, what was the name of the health or wellbeing smartphone app that you are currently using (if different from [earlier question]?	Enter free text
Would you consider using a smartphone app in the future to help you become healthier or to feel better?	(1) Yes<br>(2) No

Baseline questionnaire

Question	Response Options
What is your gender?	(1) Female<br>(2) Male<br>(3) Other [free text]

What is the highest level of education you have completed?	(1) Primary School<br>(2) GCSEs or equivalent<br>(3) A level or equivalent<br>(4) University undergraduate programme<br>(5) University post-graduate programme<br>(6) Doctoral degree
What is your employment status?	(1) Employed full-time<br>(2) Employed part time<br>(3) Self-employed full-time<br>(4) Self-employed part-time<br>(5) Unemployed<br>(6) Unemployed and on state benefits<br>(7) Unemployed - still in education
What is your ethnic group?	(1) English/Welsh/Scottish/Northern Irish/British<br>(2) Irish<br>(3) Gypsy or Irish Traveller<br>(4) Any other White background<br>(5) White and Black Caribbean<br>(6) White and Black African<br>(7) White and Asian<br>(8) Any other Mixed/Multiple ethnic background<br>(9) Indian<br>(10) Pakistani<br>(11) Bangladeshi<br>(12) Chinese<br>(13) Any other Asian background<br>(14) African<br>(15) Caribbean<br>(16) Any other Black/African/Caribbean background<br>(17) Arab<br>(18) Any other ethnic group
When was the last time you downloaded an app, if ever?	(1) Today or yesterday<br>(2) In the last week<br>(3) In the last month<br>(4) In the last 3 months<br>(5) In the last 6 months<br>(6) More than 6 months ago
How frequently do you use the apps on your smartphone, if at all?	(1) Daily<br>(2) Weekly<br>(3) Monthly<br>(4) Never
Have your friends or family recommended any smartphone health or wellbeing app for you to use?	(1) Yes<br>(2) No

Have you recommended any smartphone health or wellbeing app to your friends or family?	(1) Yes<br>(2) No
How do you use your smartphone?	(1) Check your e-mail<br>(2) For social media (e.g. Facebook, Twitter, Instagram, etc.)<br>(4) Navigate using Google Maps or similar tools<br>(5) Read the news<br>(6) Research things to purchase<br>(7) Download and play games<br>(8) Download and use health/wellbeing apps<br>(9) Other [free text]
