# Supplementary material for: Influences on the Uptake of Health and Well-being Apps and Curated App Portals: Think-Aloud and Interview Study
Source: JMIR Mhealth Uhealth. 2021 Apr 27;9(4):e27173. doi: 10.2196/27173 (PMC8114158; doi:10.2196/27173)
Supplement: Multimedia Appendix 4 [file mhealth_v9i4e27173_app4.pdf]

Multimedia Appendix 4.

Participants characteristics.

ID	Gender	Age	Ethnic group	Highest education	Employment	Health or well-being topic of interest	Ever used health apps	Current use of health app	Last time downloaded a health app	Frequency of health app use
P1	Male	28	Mixed	UG	part-time	Physical Activity, Depression, Anxiety, Mood	Yes	No	More than 6 months ago	Infrequently
P2	Female	44	British	Prof qual.	full-time	Diet, Physical Activity, Depression, Anxiety, Mood	Yes	Yes	In the last month	Daily
P3	Female	44	Other African	UG	full-time	Diet, Physical Activity, Depression, Anxiety, Mood	Yes	No	More than 6 months ago	None
P4	Male	46	British	UG	full-time	Diet, Physical Activity, Depression, Anxiety, Mood	Yes	No	More than 6 months ago	None
P5	Male	37	British	A levels	full-time	Alcohol consumption, Diet, Physical Activity, Mood	Yes	No	More than 6 months ago	None
P6	Female	53	British	PGT	full-time	Diet, Physical Activity, Depression, Mood	Yes	Yes	In the last month	Daily
P7	Male	22	Mixed	A levels	student	Physical Activity, Depression, Mood	Yes	No	In the last 6 months	Less often than monthly
P8	Male	52	British	UG	full-time	Diet, Physical Activity	Yes	Yes	In the last month	Daily
P9	Male	38	British	PGT	full-time	Diet, Depression	Yes	Yes	In the last month	Daily
P10	Female	48	British	GCSE	part-time	Diet, Physical Activity, Depression, Anxiety, Mood	Yes	No	In the last 6 months	Weekly

P11	Female	68	British	GCSE	retired	Depression, Anxiety, Mood	Yes	Yes	In the last month	Daily
P12	Male	57	British	GCSE	retired	Alcohol consumption	No	No	*Not applicable*	*Not applicable*
P13	Male	28	British	UG	full-time	Smoking, Diet, Physical Activity, Mood	Yes	Yes	In the last month	Weekly
P14	Female	64	British	GCSE	full-time	Diet	Yes	Yes	In the last 6 months	Weekly
P15	Female	56	British	GCSE	full-time	Diet, Physical Activity	Yes	Yes	More than 6 months ago	Daily
P16	Female	34	Other white	A levels	full-time	Smoking, Diet, Physical Activity, Depression, Anxiety, Mood,	Yes	Yes	In the last 3 months	Weekly
P17	Male	31	British	UG	full-time	Diet, Physical Activity, Depression	Yes	Yes	More than 6 months ago	Daily
P18	Female	21	British	A levels	full-time	Diet	Yes	Yes	In the last 6 months	Daily

Note: GSCE – General Certificate of Secondary Education (in the UK), A levels – General Certificate of Education Advanced Level (in the UK); UG – Undergraduate Degree; PGT – Postgraduate Taught Degree
